# Generating spatiotemporal patterns of linearly polarised light at high frame rates for insect vision research

**DOI:** 10.1242/jeb.244087

**Published:** 2022-07-07

**Authors:** Jack A. Supple, Léandre Varennes-Phillit, Dexter Gajjar-Reid, Uroš Cerkvenik, Gregor Belušič, Holger G. Krapp

**Affiliations:** 1 Department of Bioengineering, Imperial College London, Royal School of Mines, Exhibition Road, London, SW7 2AZ, UK; 2 Department of Biology, Biotechnical Faculty, University of Ljubljana, 1000 Ljubljana, Slovenia

**Keywords:** Electrophysiology, Neuroethology, Neuroscience, Polarisation vision

## Abstract

Polarisation vision is commonplace among invertebrates; however, most experiments focus on determining behavioural and/or neurophysiological responses to static polarised light sources rather than moving patterns of polarised light. To address the latter, we designed a polarisation stimulation device based on superimposing polarised and non-polarised images from two projectors, which can display moving patterns at frame rates exceeding invertebrate flicker fusion frequencies. A linear polariser fitted to one projector enables moving patterns of polarised light to be displayed, whilst the other projector contributes arbitrary intensities of non-polarised light to yield moving patterns with a defined polarisation and intensity contrast. To test the device, we measured receptive fields of polarisation-sensitive *Argynnis paphia* butterfly photoreceptors for both non-polarised and polarised light. We then measured local motion sensitivities of the optic flow-sensitive lobula plate tangential cell H1 in *Calliphora vicina* blowflies under both polarised and non-polarised light, finding no polarisation sensitivity in this neuron.

## Abstract (precis)

**Summary:** Design of a versatile visual stimulation device for presenting moving patterns of polarised light, and demonstration of its use to characterise polarisation sensitivity in butterfly photoreceptors and blowfly motion-sensitive interneurons.

## INTRODUCTION

Light becomes polarised when scattered by small particles or reflected from a surface (Cronin and Marshall, 2011). Polarisation refers to the distribution of electric field vector orientations within a light beam. The angle of polarisation (AoP) and the degree (i.e. ratio of the polarised to total light intensity) of linear polarisation (DoLP) are dependent on the relative position of the light source and the physical structure of the polarising material (Labhart, 2016). Thus, alongside wavelength, intensity and the propagation direction of light, AoP and DoLP provide information about the physical properties of the visual environment (Labhart, 2016). Consequently, many animals exploit spatiotemporal patterns of linearly polarised light to guide tasks as diverse as navigation (Dacke, 2014; Zeil et al., 2014), communication (How et al., 2015; Mäthger et al., 2009; Sweeney et al., 2003), water seeking (Obayashi et al., 2021) and object detection (Foster et al., 2014; Kelber et al., 2001; Meglič et al., 2019).

Historically, experimental polarised light stimuli usually comprised static light sources filtered through a linear polariser which is rotated throughout the experiment. This enabled investigation of widefield polarisation cues for navigation (Evangelista et al., 2014; Henze and Labhart, 2007; Mappes and Homberg, 2004; Mathejczyk and Wernet, 2019; Warren et al., 2018; Weir and Dickinson, 2012), polarisation-sensitive photoreceptors (Bandai et al., 1992; Blum and Labhart, 2000; Weir et al., 2016; Zufall et al., 1989) and interneurons (Hardcastle et al., 2021; Heinze and Homberg, 2007; Träger and Homberg, 2011; Zittrell et al., 2020). However, this design is less suited for investigating the role of structured, spatiotemporal patterns of polarisation contrast in object detection and motion vision.

More recent solutions for generating moving patterns of polarisation contrasts involve modifying liquid crystal display (LCD) monitors, and have been used to investigate optomotor reflexes (Drerup and How, 2021; Glantz and Schroeter, 2006) and object detection (How et al., 2014; Pignatelli et al., 2011; Temple et al., 2012). Depending on the LCD style, voltages applied across individual liquid crystal pixels change either the AoP (twisted-nematic LCDs) or resultant DoLP [patterned vertical alignment (PVA) LCDs] (Foster et al., 2018). Whilst modified LCDs are unable to control intensity contrast in addition to polarisation control, inclusion of rear-projected images from a digital light processing (DLP) projector onto PVA-style LCDs has been used to independently control intensity and DoLP contrasts (Drerup and How, 2021; Smithers et al., 2019). Changes in AoP are possible by physically rotating the system; however, the frame rate is limited by the LCD monitor (typically 60–120 Hz). An alternative method combining control of intensity, AoP and DoLP utilised a DLP projector to display video frames through a synchronously rotating linear polariser, relying on the low-pass temporal filtering properties of photoreceptors to integrate each pattern into a resultant polarisation-contrast image (Stewart et al., 2017). However, the AoP resolution and frame rate of moving patterns were limited by the rotation speed of the polariser.

**Fig. 1.**
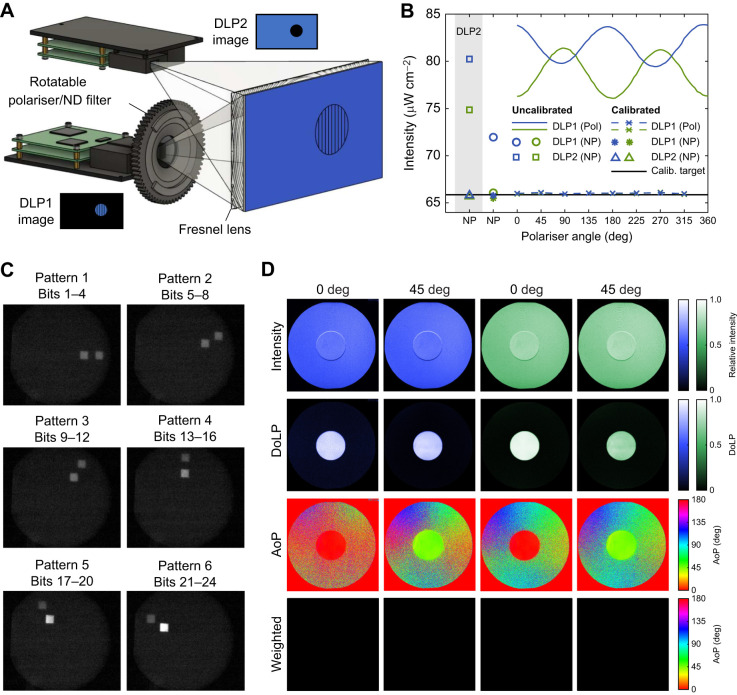
**Dual** **digital light processing (DLP)** **stimulation device for projecting moving patterns of polarised light.** (A) Images from two DLP projectors are aligned and superimposed. Patterns can be projected with polarised intensity contrasts (DLP1) or non-polarised intensity contrasts (DLP2). Superimposing inverted images from the two DLPs nullifies intensity contrasts. Polariser and neutral density (ND) filters can be exchanged for control experiments. (B) LED luminance equalisation of the two projected images for blue and green light. Polarised (Pol) DLP1 intensities varied sinusoidally with polariser angle as a result of intrinsic DLP polarisation. The lowest uncalibrated luminance [DLP1 green non-polarised (NP)] was designated the calibration target (Calib. target) to which other intensities were matched by adjusting LED currents. (C) To verify temporal synchrony, two squares, one from each projector, were filmed moving along circular trajectories with offset radii. The six panels correspond to the motion of each square along its circular trajectory at 360 Hz. Angular correspondence of each square position indicates that the two projectors are synchronised. (D) Polarimetry of the dual DLP system displaying a polarised dot at 0 deg and 45 deg angle of polarisation (AoP) against a luminance-equalised background of the same colour. From top to bottom: normalised total intensity; degree of linear polarisation (DoLP); AoP; AoP with pixel brightness weighted by the DoLP (low DoLP pixels appear darker than higher DoLP pixels).

In many insects, the flicker fusion frequency, i.e. the frequency at which photoreceptor responses no longer discriminate between sequential contrast changes, is between 100 and 300 Hz (Chatterjee et al., 2020; Cosens and Spatz, 1978; Ruck, 1961; Tatler et al., 2000). To generate stimuli that exceed these flicker fusion frequencies, we designed a stimulation device based on superimposing polarised and non-polarised images from two high frame rate DLP projectors (Fig. 1A). The superposition of image from the two DLPs enables the presentation of visual motion stimuli salient in either intensity-only contrast or polarisation-only contrast, or a combination of both intensity and polarisation contrasts. This design enables the DLPs to project motion images at high frame rates, delegating control of polarisation contrast to a dedicated projector.

To demonstrate the performance of the device, we measured receptive fields of *Argynnis paphia* butterfly photoreceptors for both non-polarised and polarised light. Butterfly blue and green photoreceptors are maximally sensitive to light polarised along the dorsal–ventral and medial–lateral axes, respectively, with a polarisation sensitivity ratio of ∼2 (Bandai et al., 1992; Kelber et al., 2001). We measured the receptive fields of these polarisation-sensitive photoreceptors to confirm the operation of the dual DLP polarisation device.

We then used the device to characterise the receptive field of the lobula plate tangential cell (LPTC) H1 in the blowfly *Calliphora vicina* for non-polarised and polarised light. LPTCs are a class of interneurons in the lobula plate that integrate local motion across large regions of visual space, resulting in receptive fields selective for specific patterns of optic flow (Borst et al., 2010; Franz and Krapp, 2000; Hausen, 1984). H1 is a spiking LPTC sensitive to horizontal back-to-front optic flow across the ipsilateral eye equator, and projects to LPTCs in the contralateral lobula plate (Hausen, 1976; Horstmann et al., 2000; Krapp et al., 2001; Weber et al., 2010). The multiplication of time-delayed responses from neighbouring ommatidia renders the insect elementary motion detector dependent on the square of the intensity contrast (Buchner, 1976; 1984). Consequently, LPTCs are highly sensitive to changes in image contrast, making them well suited to test for intensity contrast artefacts in the superimposed, intensity-masked DLP images.

A subset of LPTCs synapse with descending neurons that control optomotor stabilisation reflexes in response to optic flow (Haag et al., 2007; Suver et al., 2016; Wertz et al., 2009a; 2009b). The optomotor response is driven primarily by broadband spectral sensitivity of R1–R6 photoreceptors (Yamaguchi et al., 2008). However, R7–R8 photoreceptors have been found to improve optomotor-related motion detection via electrical coupling with R1–R6 photoreceptors in *Drosophila* (Shaw et al., 1989; Wardill et al., 2012). In *Tabanus*, a subset of R7–R8 photoreceptors within pale-type ommatidia comprise a blue–UV polarisation analyser (Meglič et al., 2019). Thus, if this R7–R8 polarisation analyser is present among other dipterans, we may expect to find polarisation sensitivity in LPTCs. However, in this study, we found no evidence for polarisation sensitivity of the H1 cell in *C. vicina*.

## MATERIALS AND METHODS

### Dual-DLP stimulation device

Two LightCrafter 3000 DLPs (Texas Instruments, Dallas, TX, USA) were mounted on a 20×20 mm aluminium extrusion frame, projecting through a polarisation-preserving rear-projection screen (ST-Pro-X, Screen-Tech e.K., Hohenaspe, Germany). A Fresnel lens (Milaosk, Moxs) was positioned between the projector and projection screen to collimate light from each DLP, reducing intensity gradients across each projected image. Note, that our original choice of Fresnel lens introduced an increased modulation of DoLP with AoP (see Fig. S1D; see also ‘Polarimetry’, below). However, this artefact can be minimised (Fig. S1D) by using a thicker acrylic Fresnel lens (280×280×2 mm, focal length 220 mm). The top DLP (DLP2) was rotated upside-down and vertically displaced from the lower DLP (DLP1) until the projected images were superimposed (Fig. 1A). To precisely align the two projectors, the top DLP was attached to the frame via a custom four-screw spring platform enabling adjustments in roll, pitch and up-down translation (Fig. S1A,B). Sideways and back-to-front translation and yaw rotation were enabled by a circular attachment of the spring platform to the extrusion beam (Fig. S1B).

The bottom DLP1 was fitted with a 3D printed rotation mount (https://www.thingiverse.com/thing:5224352) controlled by an Arduino Nano, A4988 driver and stepper motor (Nema 17HS4023). Small cylindrical neodymium magnets (6 mm diameter×2 mm, first4magnets, Tuxford, UK) on the rotation mount enabled exchange of either a linear polariser (LPVISE2X2, Thorlabs, Ely, UK) or a non-polarising neutral density (ND) filter (Thorlabs, product NE05B). The top DLP2 was fitted with a fixed non-polarising ND filter (Thorlabs, product NE05B) to roughly equalise the luminance of the two DLPs. DLP intensities were precisely equalised by adjusting the current supply to each DLP LED (Fig. 1B). Projection intensities were measured with a photodiode (Stock no. 642-4430, RS Components Ltd, Corby, UK) calibrated by a photometer (AccuPRO XP-4000 Plus, Spectronics Corporation, Melville, NY, USA). Because of intrinsic polarisation of the system, LED luminance varied sinusoidally with rotation of the polariser (Fig. 1B). This was compensated for by selecting the lowest uncalibrated luminance for all LEDs under both non-polarised and polarised conditions as the calibration target. An automated gradient descent algorithm adjusted each LED current to reach this luminance target, and this was then stored as a polariser angle-dependent look-up table for each LED.

The two DLPs were connected to the display ports of an AMD RX 580 graphics card controlled by a ROG STRIX x470-f gaming motherboard, Ryzen 5 3600x CPU and Corsair Vengeance LPX 2×8GB DDR4 3200 MHz RAM. DLPs were configured as an extended ‘Eyefinity display’ video wall with V-Sync enabled using the AMD Adrenaline graphics driver. Images were streamed to each DLP as 60 Hz 24-bit RGB .GIF videos using StimulateOpenGL (StimGL) II v.20160216 (https://github.com/cculianu/StimulateOpenGL_II). Each DLP was configured to parse the 24-bit RGB images in BRG order as 4-bit-depth sequences per pixel (i.e. greyscale values of 0–15) to yield a moving pattern frame rate of 360 Hz. DoLP is adjustable by varying the relative intensity of each projector across this 4-bit-depth range, ensuring that the summed greyscale values per pixel equal 15. For example, maximal DoLP occurs when DLP1=15, DLP2=0; minimal DoLP when DLP1=0, DLP2=15 (Fig. S1E,F). Temporal synchrony of the two projectors was verified by high-speed videography (1000 frames s^−1^; SA3 Fastcam, Photron, West Wycombe, UK), filming two test targets, one from each projector, moving along synchronised circular trajectories with offset radii (Fig. 1C). Visual stimuli were synchronised with electrophysiology traces via a photodiode sensing a small 50×50 pixel box alternating black and white every frame. Scripts to calibrate and operate the dual DLP device are available from GitHub (https://github.com/jacksupple/PolarisationStimulationDevice.git).

### Polarimetric imaging

Polarimetry of the dual DLP setup demonstrated the confinement of linear polarisation to the polarised DLP (Fig. 1D). The DoLP reached maximum values of 0.94 and 0.92 for green and blue, respectively. Note that the DoLP varied sinusoidally with AoP, reaching a maximum at AoP=[0 deg,90 deg] for both colours (Fig. S1D). This artefact arose from variable polarisation preservation, dependent on incident AoP through the rear projection screen and Fresnel lens (see Fig. S1D). Whilst the variation in DoLP due to this artefact is small (6–8%), care should be taken when comparing the polarisation sensitivities of responses with AoP preferences offset between 0 and 90 deg.

Images were calculated from a series of digital photographs taken through a camera-mounted circular polariser filter (i.e. distinct from the linear polariser mounted on the polarised DLP) with the angle of polarisation rotated in 45 deg increments using an automated polarimeter attachment (https://www.thingiverse.com/thing:5220854). Photographs were acquired on a tripod-mounted Canon 7D DSLR in manual mode (aperture f/8, shutter speed 1/10 s, ISO 100) using a 50 mm prime lens (Canon EF 50 mm f/1.8 STM) with an attached circular polariser filter (Hoya Pro1 Digital Filter, Tokyo, Japan). Images were acquired as .CR2 raw image files, then reformatted as 16-bit, un-brightened and un-gamma-corrected TIFF files using DCRAW software (https://www.dechifro.org/dcraw/) to preserve linearity between camera sensor readings and light intensity (Sumner, 2014). Bayer mosaic RGGB colour channels were kept separate, with the blue and a single green Bayer channel selected for analysis of the blue and green LEDs, respectively. AoP and DoLP were calculated from Stokes parameters *S*_0_–*S*_2_ as described previously (Foster et al., 2014, 2018):
(1)

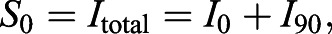

(2)

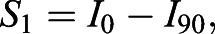

(3)

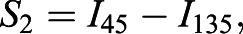
where *I*_0_–*I*_135_ are the per pixel intensity measurements with the camera polariser positioned at 0 deg to 135 deg, respectively. The AoP was calculated as:
(4)

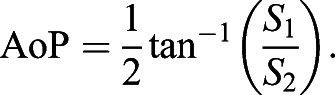
The DoLP was calculated as:
(5)

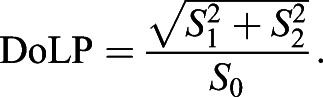


### Butterfly photoreceptor electrophysiology and receptive field mapping

*Argynnis paphia* (Linnaeus 1758) butterflies were wild caught in Ljubljana, Slovenia, in August 2021. Six photoreceptors from three *A.* *paphia* adult males were recorded intracellularly using sharp borosilicate microelectrodes, filled with 3 mol l^−1^ KCl (*R*=80 MΩ). Recording electrodes were advanced through a small hole cut in the dorsal aspect of the eye; a 50 μm Ag/AgCl wire in the opposite eye served as a reference. Details of preparation and positioning are described elsewhere (Belušič et al., 2021). Signals were amplified in bridge mode through a SEC-10LX amplifier (NPI, Tamm, Germany) and digitised at 30 kHz on a USB-6215 DAQ (National Instruments, Austin, TX, USA). Data were collected and analysed in MATLAB.

First, photoreceptor spectral sensitivity was determined using flash stimulation from a LED spectral array, combined with a diffraction grating (Belušič et al., 2016, 2021). Photoreceptor receptive fields were then mapped by measuring membrane depolarisations in response to a series of 400 small (0.5 deg×0.5 deg) stationary square objects presented for 100 ms at adjacent locations on a 20×20 grid, with 50 ms delay between each square. Stimuli comprised bright non-polarised squares on a dark background (Weber contrast: +1), bright polarised squares on a dark background (Weber contrast: +1) and polarised squares on a bright non-polarised background (Weber contrast: 0; Fig. 2). 2D photoreceptor receptive fields for each condition were calculated from the mean photoreceptor voltage during object presentation relative to the baseline membrane potential for each stimulated 2D location (Fig. S2A,B). Receptive fields were interpolated with a 2D cubic spline, smoothed with a one standard deviation 2D gaussian kernel, and normalised to the maximum response for each cell across stimulus trials (Fig. S2C,D). For each cell, receptive field *xy* displacements were calculated from the non-polarised condition as the centre of mass of binary receptive fields thresholded at 30% of the maximum value. All receptive fields were offset by this non-polarised centroid coordinate prior to averaging. Polarisation tuning curves were calculated from the maximum receptive field responses (Fig. S2C,D). Polarisation sensitivity ratios were calculated as the ratio between the maximum and minimum of the polarisation tuning curve.

**Fig. 2.**
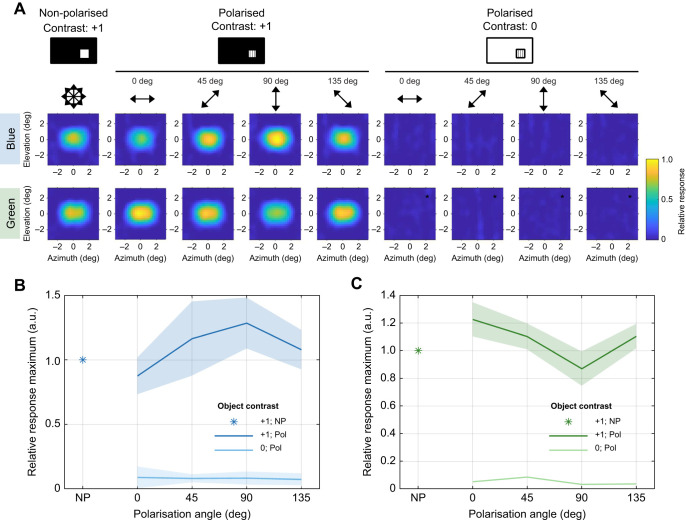
***Argynnis paphia* photoreceptor receptive fields for polarised and non-polarised light.** (A) Receptive fields of polarisation-sensitive blue and green photoreceptors. Receptive fields were measured using non-polarised and polarised bright squares on a dark background (contrast=+1), and polarised objects masked with a bright non-polarised background (contrast=0). Blue: *N*=3 cells from 2 animals; green: *N*=3 cells from 2 animals, except for masked polarised objects (*N*=1; asterisks). (B) Receptive field response maxima (see Fig. S2) for blue-sensitive photoreceptors, normalised to non-polarised (NP) receptive fields (asterisk). Symbols/lines represent the mean of single trials per cell across animals; shaded regions represent 1 s.d. (C) Receptive field response maxima for green-sensitive photoreceptors, as in B. Note *N*=1 for masked polarised objects (contrast: 0), so there is no s.d.

### LPTC electrophysiology and receptive field mapping

*Calliphora* *vicina* Robineau-Desvoidy 1830 were acquired from a laboratory colony reared at 23°C and 60% humidity on a diet of water, sugar cubes and pork liver. Nine *C. vicina* adult males were mounted on a motorised mini-goniometric recording platform (mini-GRP) (J. V. Huang, Y. Yang and H. G. Krapp, in preparation) 23 cm from the dual DLP projection display. A sharp 3 MΩ tungsten electrode (UEWSHGSE3P1M, FHC Ltd, Bowdoin, ME, USA) was inserted into the exposed right-hand side lobula plate from the posterior aspect of the head capsule. Extracellular electrophysiological signals were amplified through a non-inverting operational amplifier at ×10k gain (Huang and Krapp, 2013), and digitised at 30 kHz on a NI USB-6215 DAQ (National Instruments). Data were collected in MATLAB (MathWorks, Natick, MA, USA). Extracellular action potential (spike) times were detected offline in Spike2 software (CED, Cambridge, UK) using a manually adjusted voltage threshold. Clusters of spike units were isolated manually after projecting the spike waveforms onto their first three principal components. In all cases, a single unit was detectable in the raw trace.

**Fig. 3.**
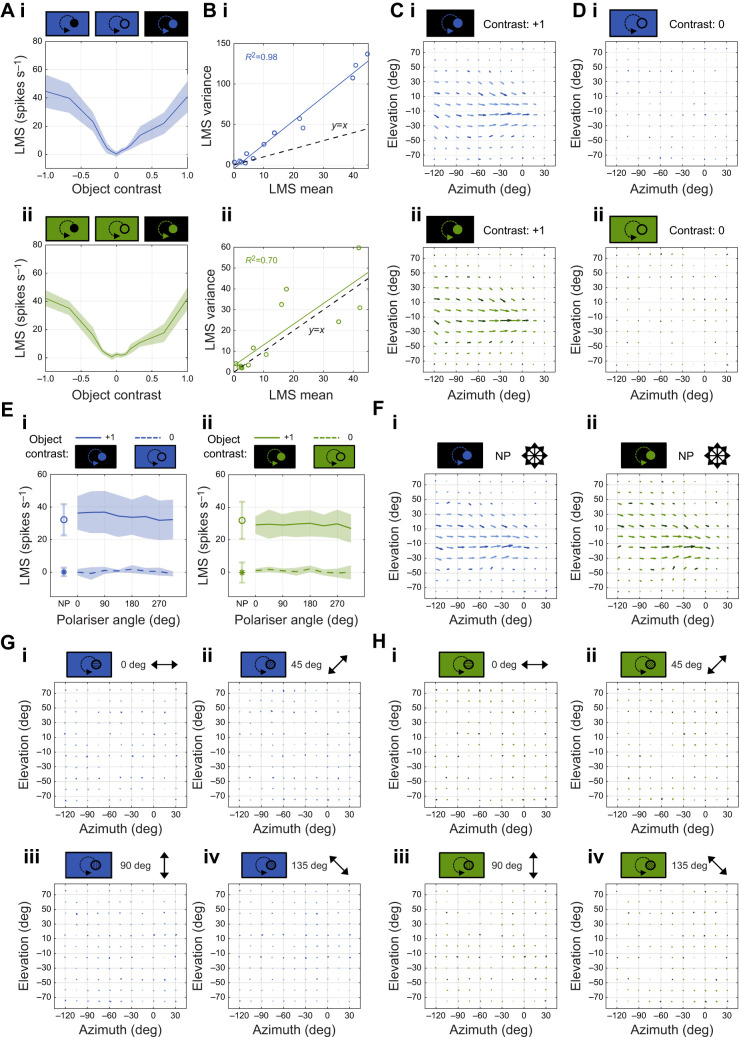
***Calliphora vicina* H1 cell motion responses to contrast artefacts and polarised light.** (A) H1 cell non-polarised control contrast tuning curves for (i) blue and (ii) green light. Both projectors are non-polarised. Contrast is varied from a dark object/bright background (Weber contrast=−1), to a bright object/bright background (Weber contrast=0, i.e. intensity-nullified control condition), to a bright object/dark background (Weber contrast=+1). Data are means±1 s.d., *N*=7 animals. (B) H1 cell non-polarised local motion sensitivity (LMS) spike rate variance plotted against LMS mean for (i) blue and (ii) green light (same data as in A). Black dashed line represents *y*=*x*. (C) Average H1 cell receptive fields for non-polarised bright (contrast=+1) (i) blue and (ii) green moving dots. Positive elevation values correspond to the dorsal visual field. Positive azimuth values correspond to the right visual field. Vector direction represents local preferred direction (LPD), vector length represents relative LMS. Dark vectors represent sampled positions; light vectors are interpolated. Blue: *N*=6 animals; green: *N*=5 animals. (D) Average H1 cell receptive fields for non-polarised and intensity contrast-nullified (contrast=0) (i) blue and (ii) green moving dots (same format as in C). Blue: *N*=6 animals; green: *N*=5 animals. (E) H1 cell polarisation tuning curves for (i) blue and (ii) green light. Bright (contrast=+1) and intensity contrast-nullified (contrast=0) dots were presented for non-polarised (NP) and polarised conditions at 45 deg AoP increments. 0 deg AoP aligns with the eye equator. Circle and solid line represent contrast=+1. Asterisk and dashed line represent contrast=0. Data are means±1 s.d., *N*=6 animals. (F) Average H1 cell receptive fields of cells (same as those in G and H) when stimulated with non-polarised bright dots (contrast=+1) for (i) blue and (ii) green light. Vector plot as in C. *N*=4 animals for each spectral condition. (G,H) Average H1 cell receptive fields of cells in F when stimulated with polarised and intensity contrast-nullified (contrast=0) dots at 45 deg AoP increments for (G) blue and (H) green light. Vector plot as in C. *N*=4 animals for each spectral condition.

LPTC local motion sensitivities (LMS) and local preferred directions (LPD) were calculated using a fast stimulation protocol (Krapp and Hengstenberg, 1997) (Fig. S3). A 7.6 deg circular dot rotated along a circular path of 10.4 deg diameter at 2 cycles per second for 10 cycles, repeated clockwise and counter-clockwise to cancel phase shifts introduced by the neuronal response latency. LPD was calculated as tangent to the circular mean of spike-triggered angular positions for clockwise (LPD_CW_) and counter-clockwise (LPD_CCW_) dots separately (Fig. S3D). Because of the delay in the neuronal response, spikes occur at a slight later phase in the stimulus after the preferred direction of motion (Krapp and Hengstenberg, 1997). The latency-corrected LPD was calculated as the resultant vector of LPD_CW_ and LPD_CCW_ (Fig. S3D). LMS was calculated from clockwise and counter-clockwise dot responses as the mean difference in spike rate ±45 deg from the LPD (*a*_CW_ and *a*_CCW_; Fig. 3D) and ±45 deg from the anti-LPD (*b*_CW_ and *b*_CCW_) (see Fig. S3D):
(6)

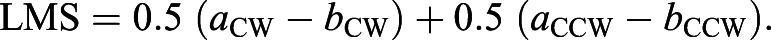
Non-polarised contrast tuning curves (Fig. 3A) were measured by varying the greyscale intensities of the moving dot and background, from a dark dot on a bright background (Weber contrast=−1), through to a bright dot on a bright background (Weber contrast=0), to a bright dot on a dark background (Weber contrast=+1). Polarisation tuning curves (Fig. 3E) were measured for contrasts of 0 (bright dot on a bright background) and +1 (bright dot on a dark background) for 45 deg AoP increments through two full rotations of the polariser. LMS values were first averaged over trials within individual animals, then averaged across animals. Both non-polarised control contrast tuning curves and polarisation tuning curves were measured at a single location corresponding to the receptive field location with maximum LMS.

All LPTC receptive fields (Fig. 3C,D,F–H) were characterised by measuring the LMS and LPD across a series of positions within the left and frontal visual field extending from −120 deg to +30 deg azimuth, and −75 deg to +75 deg elevation. Flies were automatically rotated between each stimulus presentation using the motorised mini-GRP. Vector fields were interpolated up to 15 deg increments across the sampled spatial range using a cubic spline. Receptive field vectors were normalised to the maximum value in the non-polarised condition prior to averaging across animals.

## RESULTS AND DISCUSSION

### *Argynnis paphia* photoreceptor receptive field characterisation with polarised light

To test whether the device elicits polarisation-sensitive neuronal responses, we recorded intracellularly from polarisation-sensitive photoreceptors in the retina of three *A. paphia* adult male butterflies (Fig. 2). Polarisation-sensitive *A.* *paphia* photoreceptor receptive fields resembled 2D-gaussians for both non-polarised and polarised objects on dark backgrounds (Fig. 2A). Peak receptive field responses were maximal for an AoP of 90 deg (i.e. dorsal–ventral axis) and 0 deg (aligned with the eye equator) for blue- and green-sensitive photoreceptors, respectively (Fig. 2B,C). Polarisation sensitivity (PS) ratios were 1.5±0.2 (*N*=3) for blue photoreceptors and 1.4±0.4 (*N*=3) for green photoreceptors. Response magnitudes for non-polarised objects were intermediate between the maximal/minimal polarisation responses (Fig. 2B,C), consistent with intermediate rhabdom photon capture for non-polarised light compared with polarised light of equal luminance. Overall, the dual-DLP polarisation display is sufficient to elicit polarisation-dependent responses in butterfly photoreceptors.

Photoreceptors did not respond to polarised objects on a non-polarised intensity-masked background (Fig. 2A–C), implying that photon capture contrast between a polarised object and a non-polarised background of equal luminance is insufficient to stimulate changes in photoreceptor potential. This suggests that moving patterns of polarisation-only contrast remain invisible, and some combination of polarisation and intensity contrasts is required for polarisation vision. Indeed, this agrees with the proposed integration of polarisation and intensity cues determined from the horsefly retina (Meglič et al., 2019). However, it remains possible that small photoreceptor depolarisations could be spatiotemporally pooled, with downstream neurons averaging and correlating responses across the retina.

### *Calliphora vicina* H1 cell is insensitive to superimposed projection artefacts

Despite our best efforts to align the two superimposed images and equalise screen luminance, contrast artefacts between the two images remain detectable by the human eye (Fig. 1D). Whilst this could be reduced by modifying the DLP light path (Fig. S1C), we tested whether the artefact was detected by insects by recording extracellular responses from *C.* *vicina* H1 cells under a non-polarised control condition in which both DLPs were fitted with ND filters (Fig. 3A–D). Superimposed image contrasts were varied from a dark object/bright background (contrast=−1), to a bright object/bright background (contrast=0), to a bright object/dark background (contrast=+1). At 0 contrast, the two DLP images should cancel out, resulting in a featureless projection. Neuronal responses evoked under these conditions therefore arise from intensity contrast artefacts (e.g. mismatched luminance or positional misalignment between DLPs).

H1 cell LMS contrast tuning curves were V-shaped, reaching a minimum LMS of 0.2±1.9 spikes s^−1^ at 0 contrast for blue light (*N*=7; Fig. 3Ai) and 0.7±2.0 spikes s^−1^ at −0.07 contrast for green light (*N*=7; Fig. 3Aii). LMS variance increased linearly with mean LMS, albeit at a higher rate for blue compared with green light (Fig. 3B). H1 cell receptive field structures agreed with the LPD and LMS distributions previously obtained using electromechanical stimuli (Krapp and Hengstenberg, 1997; Krapp et al., 2001). There were no differences between H1 cell receptive fields when stimulated with non-polarised blue or green light (Fig. 3C). There were no responses across the H1 cell receptive field region in the non-polarised intensity contrast-nullified condition for both blue and green light (Fig. 3D), suggesting that contrast artefacts were undetectable across the H1 cell receptive field.

The steeper rate of LMS variance increase with LMS mean for blue light (Fig. 3B) suggests that motion vision is noisier when limited to this spectrum. This may reflect input from noisy blue-sensitive photoreceptor channels, which is only detectable when stimulated with blue light. In Diptera, pale ommatidia R8 (R8p) photoreceptors express blue-sensitive Rh5 opsin, and have noisier receptor potentials compared with R1–6 photoreceptors (Meglič et al., 2019). In *Drosophila*, R7–R8 photoreceptors form electrical synapses with R1–R6 photoreceptors, contributing to motion vision (Wardill et al., 2012). R8p are therefore candidates for this additional noisy input to the H1 cell, supporting the hypothesis that multiple spectral inputs converge on dipteran motion vision pathways.

### *Calliphora vicina* H1 cell is insensitive to the angle of polarised light

To test *C.* *vicina* H1 cell polarisation sensitivity, we presented bright (contrast=+1) and intensity contrast-nullified (contrast=0) moving dots at 45 deg AoP increments in the centre of the receptive field. LMS responses were not modulated by the AoP in either contrast condition (Fig. 4E). Similarly, there were no responses to intensity contrast-nullified polarised dots when presented throughout the H1 cell receptive field (Fig. 4G,H), suggesting that the *C.* *vicina* H1 cell is insensitive to polarised light.

The polarisation insensitivity of *C.* *vicina* H1 cells implies that (i) *Calliphora* photoreceptors are insensitive to polarised blue or green light; and/or (ii) polarisation-sensitive photoreceptors do not contribute to H1 cell motion sensitivity. In *Tabanus*, R8p photoreceptors are responsible for blue light polarisation sensitivity (Meglič et al., 2019). The contribution of R8–R7 to motion vision in *Drosophila* (Wardill et al., 2012), in addition to our putative finding that R8p contributes to H1 cell motion sensitivity (Fig. 3B), suggests that the H1 cell receives input from multiple photoreceptor types. Therefore, H1 cell polarisation insensitivity probably reflects photoreceptor polarisation insensitivity. Reproducing these polarisation motion sensitivity experiments in *Tabanus*, in which photoreceptor blue polarisation sensitivity is established (Meglič et al., 2019), will allow us to test this hypothesis.

### Conclusion

We have presented and tested a versatile device for projecting moving patterns of polarised light at high frame rates for insect vision, using two superimposed DLP projections. The device stimulates AoP-dependent responses in butterfly photoreceptors, and experiments in blowfly H1 cells suggest that residual contrast artefacts in the superimposed projection do not elicit motion-sensitive responses. Furthermore, no evidence was found for polarisation sensitivity in the blowfly H1 cell.

## Supplementary Material

Supplementary information
