# Supplementary material for: Generating spatiotemporal patterns of linearly polarised light at high frame rates for insect vision research
Source: J Exp Biol. 2022 Jul 7;225(13):jeb244087. doi: 10.1242/jeb.244087 (PMC9339910; doi:10.1242/jeb.244087)
Supplement: Supplementary information [file jexbio-225-244087-s1.pdf]

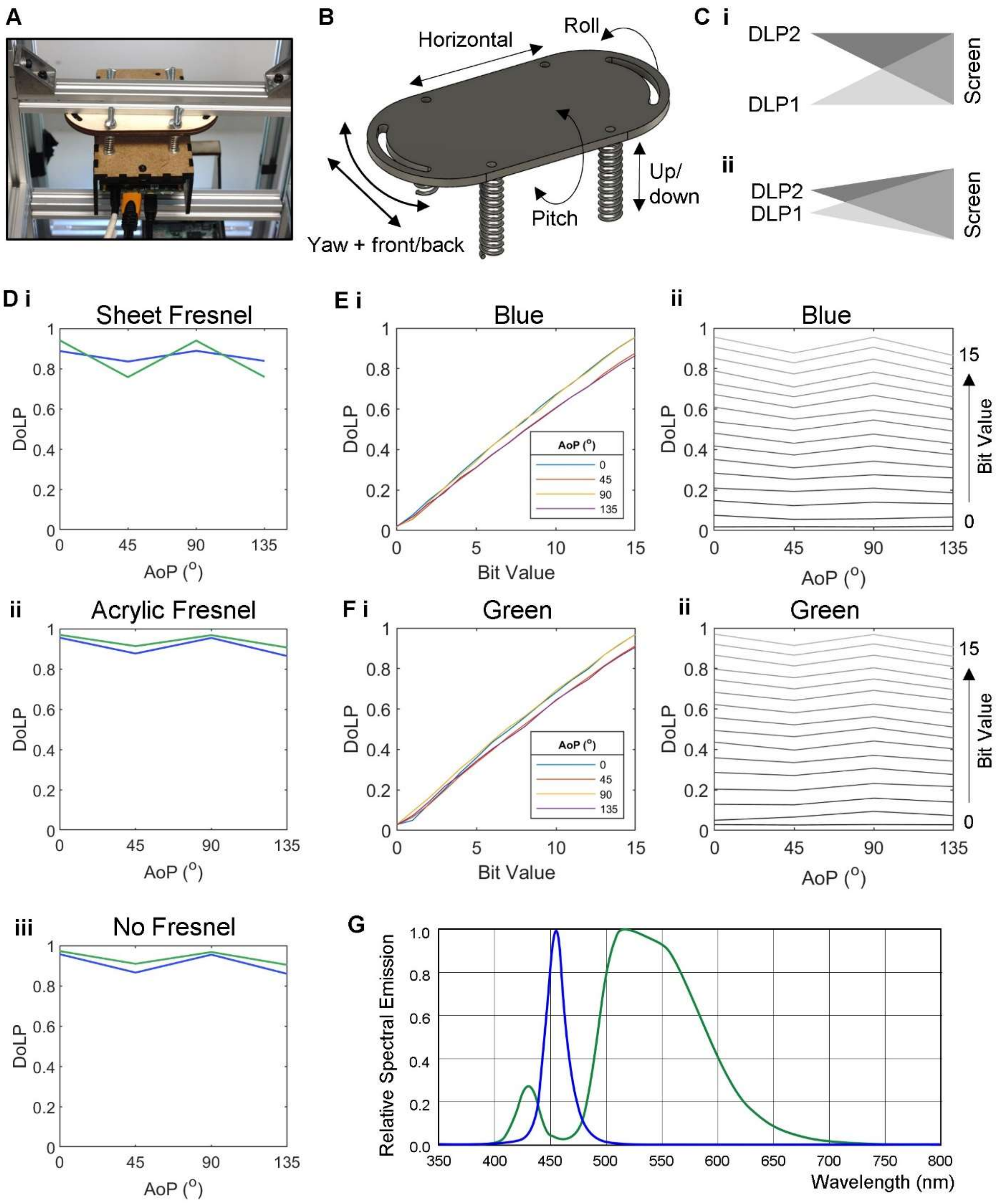

Fig. S1. Positional alignment platform for the upper DLP (DLP2). (A) Photograph of the top DLP spring platform connector. **(B)** Spring platform and connector to adjust DLP2 angle and position with 6 degrees of freedom. **(C) (i)** We used DLPs with a standard 100% image offset, in which the centre of the bottom edge of the projected image is aligned with the DLP lens axis. This results in an angle between the projection axes of each DLP, which contributes to an intensity contrast gradient artefact, as the image appears brighter when the viewer looks at the projection screen along

Journal of Experimental Biology • Supplementary information

the DLP projection axis. **(ii)** The contrast gradient could be minimised by reducing the angle between each DLP, for instance by shifting the internal DLP lens to produce image offsets <100%. **(D)** DoLP varies with AoP due to differential polarisation preservation and should be considered when interpreting polarisation responses. **(i)** Our original sheet Fresnel lens introduced a large amplitude DoLP vs AoP sinusoid (green DoLP min=0.76, max=0.94; blue DoLP min=0.87, max=0.92). **(ii)** The artefact was minimised but not eliminated by replacement with a thicker acrylic Fresnel lens (green DoLP min=0.91, max=0.97; blue DoLP min=0.88, max=0.96). **(iii)** The DoLP sinusoid was also present in the absence of a Fresnel lens (green DoLP min=0.91, max=0.97; blue DoLP min=0.86, max=0.96). **(E-F)** Range of DoLP available by varying the greyscale bit value of DLP1 between 0-15. The bit value of DLP2 is adjusted such that the summed greyscale values per pixel equal 15. Minimum DoLP = 0.02 and 0.03 for blue and green light, respectively. Same data in (i) and (ii). **(G)** Relative spectral emission of the blue (LE-B-Q9WN) and green (LCG-H9RN) LEDs.

Journal of Experimental Biology • Supplementary information

Journal of Experimental Biology • Supplementary information

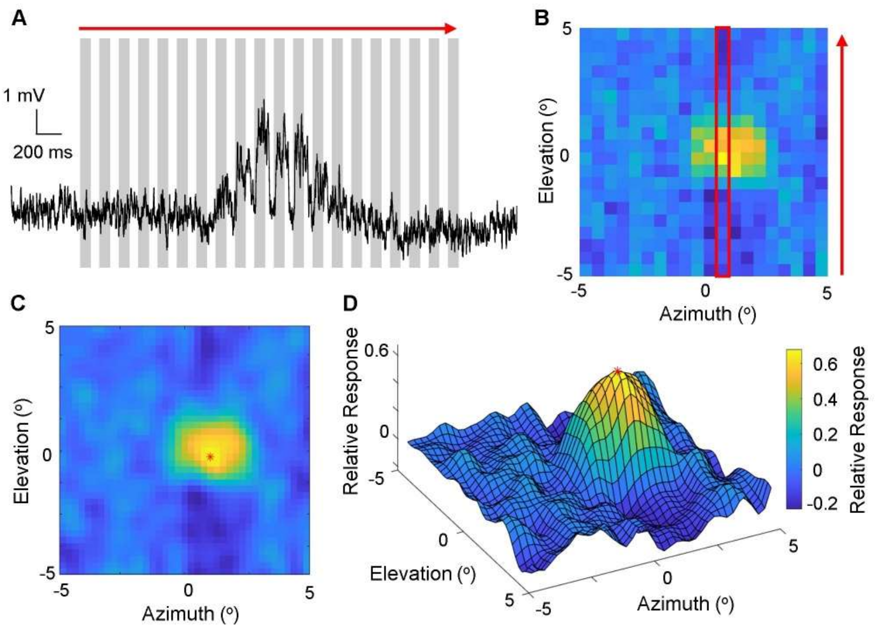

Fig. S2. Photoreceptor receptive field characterisation

(A) Intracellular photoreceptor membrane voltage during 20 flashes of a bright non-polarised blue square on a dark background, through the centre of the receptive field. Stimulus presentation indicated by grey bars. Red arrow represents the sequential series of stimuli and corresponds to the arrow in (B).

(B) Receptive field reconstructed from mean membrane potential voltages during each of 400 (20x20) presentations of the small square stimulus. Red rectangle corresponds to the 20 stimulus presentations depicted in (A). Red arrow indicates the temporal sequence of stimulus presentations, corresponding to the red arrow in (A).

(C) Interpolated and smoothened receptive field from (B). Receptive field maximum indicated by asterisk. This maximum value forms the tuning curves in Figure 2B-C.

(D) Side view of the same receptive field as in (C), receptive field maximum indicated by asterisk. Note that receptive fields are normalised to the maximum value across all polarised and non-polarised conditions. This non-polarised receptive field has a maximum value of 0.68, whilst the 90° polarised condition (not shown) is overall maximal at a value of 1.0.

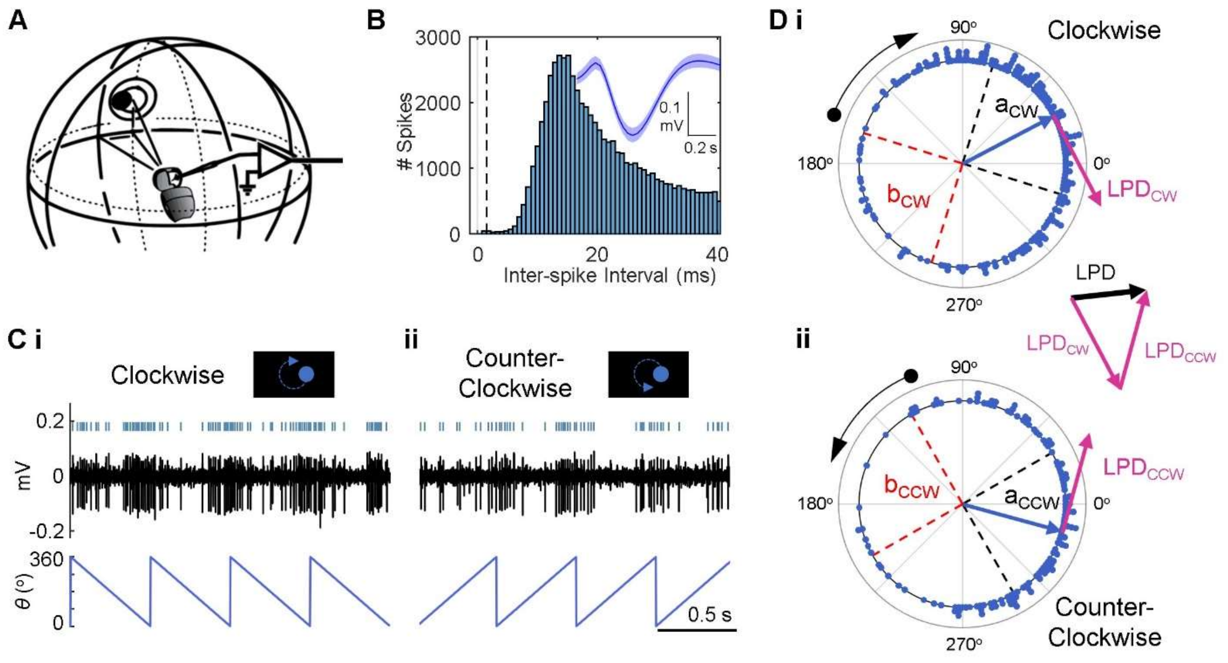

Fig. S3. Fast stimulus protocol for LPTC experiments

(A) LPTC neurons were stimulated by a small (7.6^{o} diameter) dot moving at 2 cycles/s through a circular path subtending a small (10.4^{o} diameter) region of visual space. Figure reproduced from (Huston and Krapp, 2008).

(B) Example inter-spike interval histogram and corresponding extracellular spike waveform (inset). Solid line and shaded area represent mean±1 std of 98471 spikes, respectively.

(C) Example raw electrophysiological trace of an H1-cell responding to four revolutions of a clockwise (i) and counterclockwise (ii) dot. Detected spikes indicated in blue above trace. θ represents the angular position of the dot.

(D) Spike-triggered angular positions of the dot (blue circles) for (i) clockwise and (ii) counter-clockwise dots, presented in polar coordinates. The local preferred direction (LPD) was calculated as tangent to the circular mean of spike-triggered angular positions for clockwise (LPD$_{CW}$) and counter-clockwise (LPD$_{CCW}$) dots separately. The latency-corrected LPD was calculated as the resultant vector of LPD$_{CW}$ and LPD$_{CCW}$. Local motion sensitivity (LMS) was calculated from clockwise and counter-clockwise dot responses as the mean difference in spike rate ±45° from the LPD (a$_{CW}$ and a$_{CCW}$) and ±45° from the anti-LPD (b$_{CW}$ and b$_{CCW}$, see Methods).
